# An Analysis of the Kinetic Energy in the Basket to Handstand on Parallel Bars: A Case Study of an Elite Gymnast

**DOI:** 10.3390/life15020172

**Published:** 2025-01-25

**Authors:** Saša Veličković, Dušan Đorđević, Petar Veličković, Marijo Možnik, Edvard Kolar, Cristina-Elena Stoica, Alina-Mihaela Cristuță, Nicolae-Lucian Voinea, Ana-Maria Vulpe, Saša Bubanj, Dušan Stanković, Bojan Bjelica, Nikola Aksović, Tatiana Dobrescu

**Affiliations:** 1 Faculty of Sport and Physical Education, University of Niš, 18000 Niš, Serbia; v.sale70@gmail.com (S.V.); dusandjordjevic1995@gmail.com (D.Đ.); gimnastika1997@gmail.com (P.V.); dukislavujac@gmail.com (D.S.); 2 Faculty of Kinesiology, University of Zagreb, 10000 Zagreb, Croatia; marijo.moznik@kif.unizg.hr; 3 Science and Research Centre Koper, 6600 Koper, Slovenia; edvard.kolar@zrs-kp.si; 4 Department of Physical Education and Sport Performance, Vasile Alecsandri University, 600115 Bacau, Romania; cristina.popa@ub.ro (C.-E.S.); cristuta.alina@ub.ro (A.-M.C.); lucian.voinea@ub.ro (N.-L.V.); zaharia.ana@ub.ro (A.-M.V.); 5 Faculty of Sport and Physical Education, University of Priština, Kosovska Mitrovica, 38218 Leposavić, Serbia; vipbjelica@gmail.com; 6 Faculty of Physical Education and Sports, University of East Sarajevo, 71126 Lukavica, Bosnia and Herzegovina; kokir87np@gmail.com

**Keywords:** kinetic energy, gymnastics, kinematic analysis, parallel bars, biomechanics

## Abstract

(1) Background: This study aimed to examine the differences in the kinetic energy of the body’s center of mass between successful and unsuccessful attempts at transitioning from a basket to a handstand on the parallel bars. Special attention was given to the analysis of kinetic energy as a key factor in the efficient execution of this complex element. (2) Methods: The sample consisted of 10 successful and 10 unsuccessful attempts performed by an elite gymnast (a multiple-medalist in World and European Championships). All attempts and kinematic data were recorded and analyzed using high-frequency cameras (300 Hz) and the Ariel Performance 3D video system, respectively. Successful and unsuccessful performances were compared using a paired-sample *t*-test. (3) Results: Significant differences in kinetic energy were observed in the first part of the anti-gravitational phase of movement between successful and unsuccessful attempts. Successful attempts demonstrated a more favorable position at the beginning of this phase, allowing better utilization of accumulated kinetic energy—a higher position of the feet and hips, and a smaller shoulder joint angle at the moment the shoulder passed through the lower vertical. (4) Conclusions: Successful attempts in gymnastics are characterized by better biomechanical optimization and efficient kinetic energy use, achieved through an earlier entry into the second phase of movement with optimal body positioning, leading to greater peripheral and angular velocities crucial for performance.

## 1. Introduction

The model of successful performance for competitors in gymnastics consists of highly complex coordination elements and their precise execution. A gymnast will be more successful if they perform the most advanced coordination elements, such as those of D, E, F, G, and H difficulty levels [1] (FIG), in their routines with minimal technical and esthetic errors [2].

Therefore, examining the technique of these complex elements and the errors made during their execution is a key challenge for research in gymnastics. A rational and economical process for teaching and perfecting these elements requires detailed analysis, particularly for aspects of the technique that are not easily accessible to the coach’s visual inspection or the gymnast’s kinesthetic receptors. These hidden technical details can only be revealed through biomechanical analysis, specifically through the analysis of kinetic energy.

Research in the field of biomechanical movement analysis is becoming increasingly common in gymnastics, particularly over the last few decades, with a significant increase in the number of publications since 2015 [3]. This trend reflects a continuous growth of interest in biomechanical research in gymnastics, emphasizing the importance of such analyses for optimizing performance and providing information to enable more rational and efficient training, as well as the acquisition of complex movements [4,5]. Furthermore, novel research is needed to identify and develop more effective strategies for enhancing gymnast performance. Kinematic analysis plays a crucial role in understanding and improving movement execution techniques across various sports disciplines, particularly in gymnastics, where precision and control are of utmost importance [6]. However, while kinematics is well-studied, the study of the role of kinetic and potential energy in sports, particularly in gymnastics, is still a relatively new topic.

Recent research has focused on various aspects of kinetic energy in sports. For example, Hoareau and associates [7] investigated the available sources of kinetic energy in the human body during sports activities, focusing on its potential to be converted into electrical energy using piezoelectric harvesters. This research underscores the importance of kinetic energy in developing wearable sensors for monitoring sports performance. Wasserberger and associates [8] analyzed the generation, absorption, and transfer of energy in the shoulder and elbow joints of young baseball pitchers, exploring the relationship between these energy measures and throwing velocity. Their findings highlight the importance of energy transfer in achieving high throwing velocities while reducing the risk of injury. Understanding energy transfer through the kinetic chain is crucial not only in gymnastics but also in other sports. These findings can be viewed as analogous to energy transfer in complex gymnastics elements, where the efficient use of kinetic energy can significantly impact performance and reduce the risk of injury. Similarly, Priest and associates [9] focused on evaluating athletic performance by considering athletes’ kinetic energy, which includes velocity and body mass. Thus, kinetic energy, which accounts for both velocity and mass, is a critical factor in assessing sports performance.

Furthermore, research by Jones and associates [10] demonstrated how kinetic energy could be used to evaluate training effects and compare athletes and teams. Their work employed the “run-shuttle” test with a laser timer to measure time, velocity, and kinetic energy, providing detailed insights into athlete performance across various sports (e.g., American football, soccer, basketball, and athletics). This approach can be compared with similar analyses in gymnastics, where kinetic energy plays a crucial role in the execution technique and achieving optimal performance. Regarding previously published studies in gymnastics, Schärer and associates [11] analyzed differences in kinetic energy between Tsukahara and Yurchenko vaults using 3D motion capture technology. Their findings revealed that the Tsukahara vault is characterized by greater translational kinetic energy (TKE), whereas the Yurchenko vault has greater angular kinetic energy (AKE). For more complex vaults, 5.9% more AKE is required for each additional 180° turn. This knowledge helps coaches assess athletes’ potential and direct training toward appropriate physical and technical aspects of the vault.

Further research would provide deeper insights into how kinetic energy can be utilized and analyzed in sports, contributing to a better understanding of the technical aspects of performance. In gymnastics, a detailed understanding of biomechanical differences is essential for optimizing execution techniques and improving performance through targeted training processes.

This study aimed to examine the differences in the kinetic energy of the body’s center of mass between successful and unsuccessful attempts at transitioning from a basket to a handstand on the parallel bars, performed by an elite gymnast. Special attention has been given to kinetic energy analysis as a key factor in the efficient execution of this complex element.

## 2. Materials and Methods

### 2.1. Participant

The sample consisted of one elite gymnast (age: 26; body height: 165 cm; body mass: 63 kg). The inclusion criteria required that the participant be male and a medalist at the World or European Championships. The exclusion criteria included current injuries affecting performance and a lack of consent for participation in the study. The Gymnastics Federation of Serbia approved all experimental procedures on 27 October 2023 (approval No. 11-485/23) in accordance with the Helsinki Declaration for studies on humans [12].

### 2.2. Study Design

This study employed a case study design. One elite male gymnast was recruited to analyze kinetic energy between successful and unsuccessful attempts during the basket to handstand on parallel bars gymnastic element. The authors examined the total kinetic energy (KEtotal), kinetic energy of translational motion (KEtrans), and kinetic energy of rotational motion (KErot).

### 2.3. Procedures

**Figure 1:**
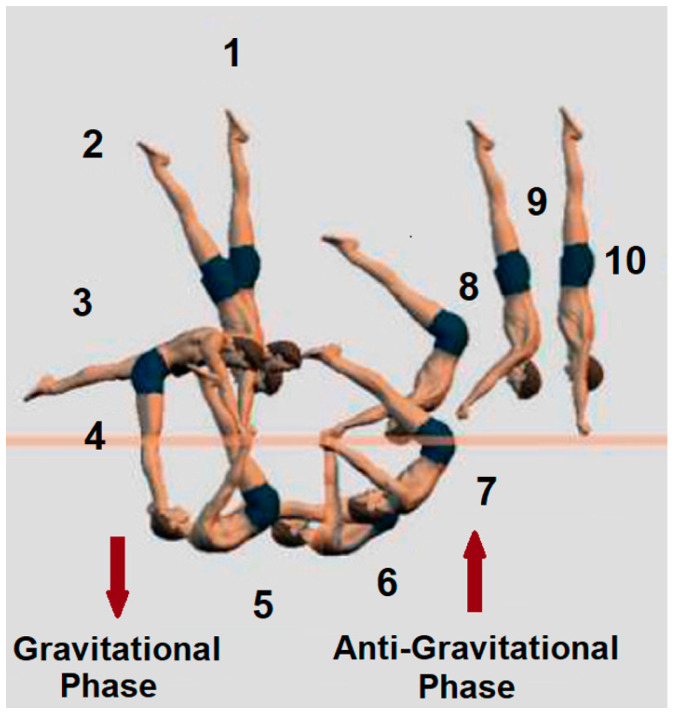
Basket to handstand on the parallel bars through positions (1–10) and phases [14].

The gymnast performed 45 attempts of the basket to handstand on parallel bars (Figure 1) under experimental conditions, with varying levels of success. Out of 45 attempts performed by the participant, 10 successful and 10 unsuccessful attempts were analyzed and graphically represented as the mean values. After each attempt, a 60 s break would follow. All repetitions were recorded using two high-frequency CASIO DIGITAL CAMERA EX-F1 devices (Casio Computer Co., Ltd., Tokyo, Japan), which were interconnected and synchronized. The cameras operated at a frequency of 300 Hz with a resolution of 720 × 576 pixels. Simultaneously, three internationally accredited judges (FIG-BREVET) evaluated the success of each attempt using the current “E” panel scoring system. The assessment focused on both technical execution, such as proper body alignment, smooth transitions, controlled movements, and maintaining straight arms, as well as esthetic elements, including pointed toes, fluidity, and overall presentation. Deductions were applied for noticeable deviations, loss of balance, or incomplete extension in the handstand position [13]. The final classification of attempts as successful or unsuccessful was determined based on the judges’ scores, adhering strictly to the FIG evaluation criteria.

**Figure 2:**
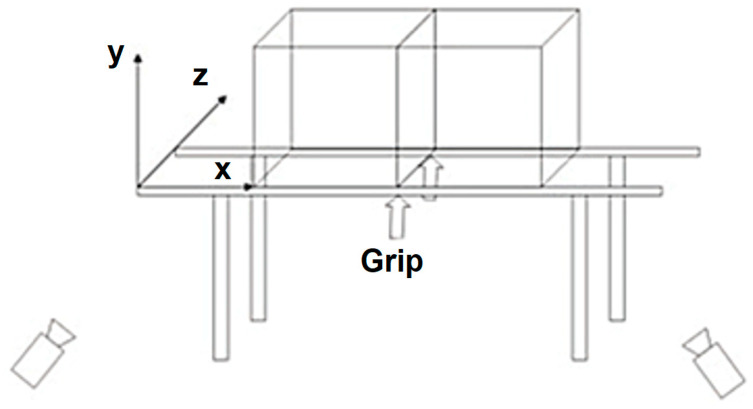
Calibration space [16].

To determine the kinematic parameters of the selected attempts, the Ariel Performance 3D analysis system (APAS, version 13.2.1) was used for kinematic analysis. The Center of Mass in the APAS is calculated using a segmental analysis approach, where each body segment’s mass and position are considered based on anthropometric data. This method integrates the positional data of body landmarks captured during motion analysis [15]. The calibration of the space was performed using two reference frames positioned in the middle of the parallel bars, allowing for precise digitalization of the gymnast’s position in each phase of the element (Figure 2). All attempts were recorded at a frequency of 300 Hz.

**Figure 3:**
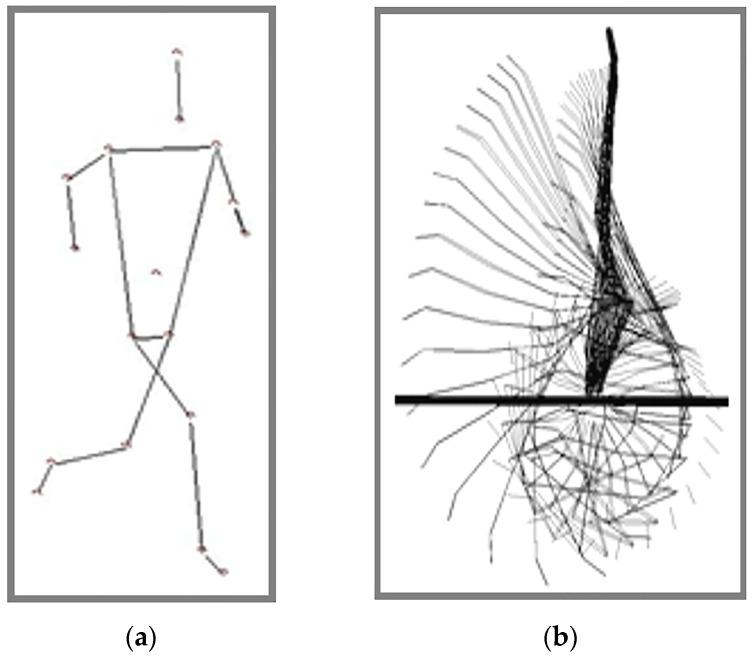
Digitizing process in APAS: (**a**) 15-segment model and (**b**) stick figure model [15].

The digitization of the athlete’s body model, consisting of 15 segments, was defined using 16 reference points [17]. The validity and reliability of the model were confirmed by [18]. As presented elsewhere [15], the 15-segment model included the head, shoulder width, left and right upper arms, left and right forearms, left and right sides of the torso, hip width, left and right thighs, left and right lower legs, and left and right feet (Figure 3a). The verification of anatomical reference positions was assessed electronically by the same rater. Two video cameras were able to record and quantitatively determine the movement of each body component. By projecting each frame of the video onto the monitor and locating the pointer, each position of the plotted reference point was assigned a numerical value from the selected coordinate system. After digitization, the APAS automatically calculated the trajectory of the body’s center of mass along the x, y, and z axes for each selected frame, as well. Since the executed element has characteristics of two-dimensional movement, only the right side of the body was considered for further analysis (Figure 3b). Additionally, as there was no significant movement along the medio-lateral (z) axis, values on the z-axis were not included in the analysis. Kinograms were presented as a stick figures, which represent simplified models of the human body and motion of the gymnast.

Following the digitization of each frame of the analyzed movement in APAS and the subsequent automatic calculation of all the necessary kinematic parameters, the kinetic energy was calculated. The formula used to calculate kinetic energy was as follows [19]:

$$\mathrm{KEtotal}=\mathrm{KEtrans}+\mathrm{KErot}=\frac{1}{2}\;{\mathrm{mv}}^{2}+\frac{1}{2}\;{I\omega }^{2}$$

Legend: KEtotal—total kinetic energy; KEtrans—kinetic energy of translational motion; KErot—kinetic energy of rotational motion; m—mass of the gymnast; v—velocity of the center of mass in translational motion; I—moment of inertia of the gymnast; ω—angular velocity of the center of mass/axis of rotation.

### 2.4. Statistical Analysis

The Kolmogorov–Smirnov test was used to assess the normality of the data distribution. To assess the difference between successful and unsuccessful attempts, a paired-samples *t*-test was used.

All statistical analyses were considered significant at *p* < 0.05. The percent difference between means were also calculated (∆ (%)). For more sensitive analysis, an effect size (ES) was calculated. The ES was presented as follows: d < 0.2—trivial effect; 0.2 ≤ d < 0.5—small effect; 0.5 ≤ d < 0.8—medium effect; and d ≥ 0.8—large effect [20]. The data were analyzed using the Statistical Package for Social Sciences (SPSS) software (v20.0, SPSS Inc., Chicago, IL, USA).

## 3. Results

In the following text, the term “positions” refers to specific moments within a basket to handstand technique. These positions mark critical points where significant biomechanical changes took place, such as alterations in the gymnast’s body posture, movement trajectory, or kinetic energy.

The analyzed element was divided into two phases [14], the gravitational and anti-gravitational phase. During the gravitational phase, the body’s center of mass moves in the direction of gravitational force and accumulates kinetic energy. Specifically, this phase includes the movement from a handstand swing (position 17) and further descent to a lifted hang (position 53). During the anti-gravitational phase, the body’s center of mass moves against the force of gravity during this phase, encompassing the transition from the lifted hang (position 53) to position 70 and continuing into the handstand.

**Figure 4:**
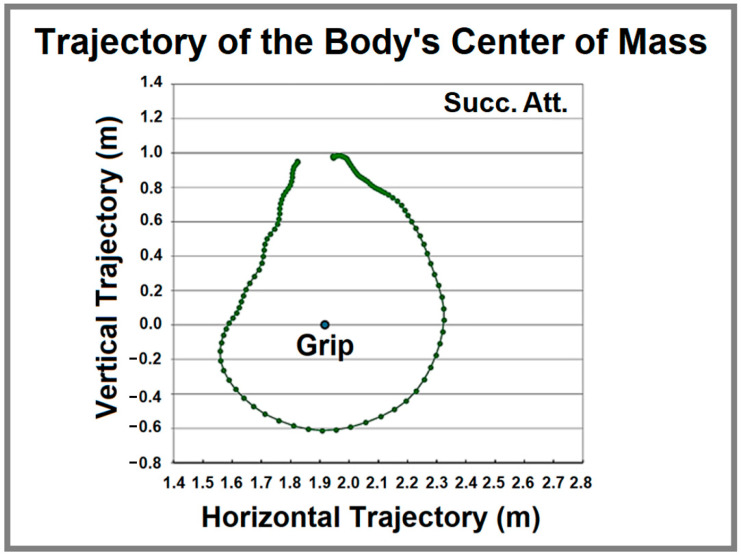
Trajectory of body’s center of mass in meters (m); Succ. Att.—figure made using mean values of successful attempts.

Notably, the entire body rotates around the grip axis (Figure 4), while additional rotations occur within subsystems, the trunk–legs system rotates around the shoulder axis, and the legs system rotates around the hip axis. The center of mass for the body was automatically calculated for each selected frame using the APAS system.

**Figure 5:**
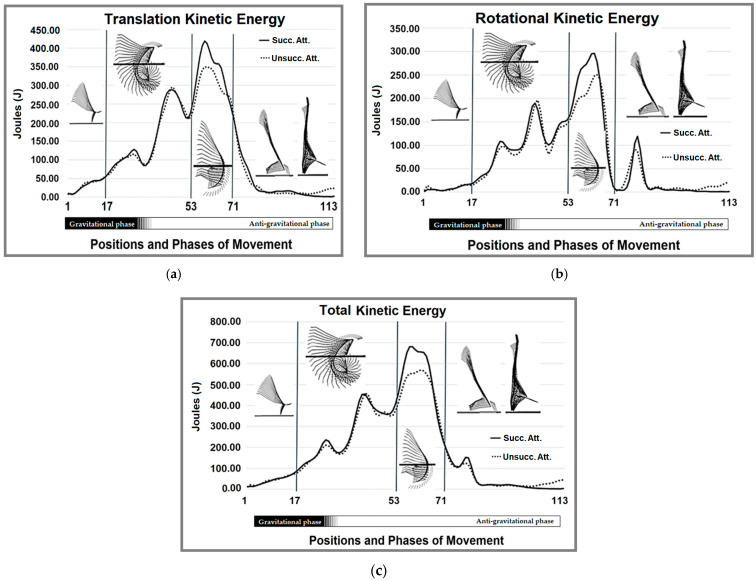
The kinetic energy of the body’s center of mass during successful and unsuccessful attempts, expressed as average values in Joules (J): (**a**) translational kinetic energy, highlighting the movement of the body’s center of mass along a linear path; (**b**) rotational kinetic energy, illustrating the rotational motion of the body’s center of mass around its axis; (**c**) total kinetic energy, which is the sum of translational and rotational kinetic energies. The *x*-axis represents the positions and phases of movement, divided into gravitational (1–53) and anti-gravitational (54–113) phases. The vertical lines indicate key transition points, with visual markers of body positions during those phases. Succ. Att.—figure made using the mean values of successful attempts; Unsucc. Att.—figure made using the mean values of unsuccessful attempts.

Figure 5a–c presents the average values of all calculated forms of kinetic energy (translational, rotational, and total) for successful and unsuccessful attempts. Regarding translational kinetic energy (Figure 5a), successful attempts demonstrate higher peaks during the anti-gravitational phase, indicating greater momentum and more effective energy transfer. Successful attempts display smoother increases for rotational kinetic energy (Figure 5b) and higher peaks during key transitions, reflecting better coordination and control of rotational movement. As for total kinetic energy (Figure 5c), successful attempts consistently achieve greater values, especially in the anti-gravitational phase, emphasizing the importance of efficiently integrating translational and rotational energies for optimal performance.

**Figure 6:**
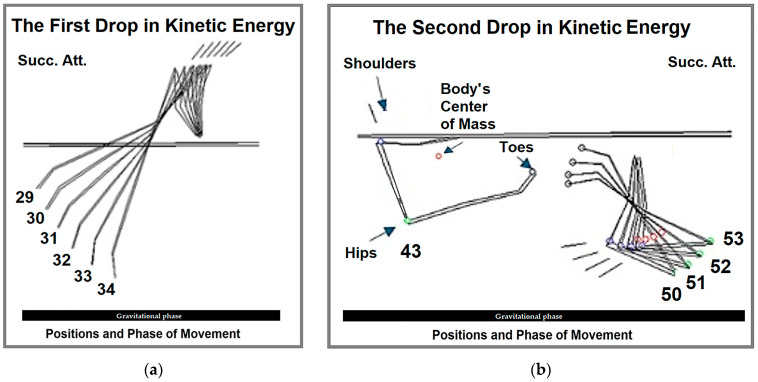
Drops in kinetic energy: (**a**) the first drop occurs between positions 29 and 34, and (**b**) the second drop occurs at position 43 and between positions 50 and 53. Succ. Att.—figure made using the mean values of successful attempts.

The kinetic energy does not increase steadily but instead fluctuates in a wave-like pattern. The first significant drop in energy occurs at position 29 (Figure 6a), coinciding with the shoulder point leaving the support surface and an increase in the shoulder joint angle due to anteflexion. This decrease is likely deliberate, as the gymnast consciously decelerates to prepare more precisely for the subsequent phase. Once the tips of the feet enter the anti-gravitational phase at position 34, dynamic hip joint flexion intensifies, leading to a renewed increase in kinetic energy.

The second drop in kinetic energy begins after position 43 (Figure 6b), as the shoulder point enters the second quadrant of its circular trajectory, moving below the bars. During this phase, the tips of the feet start ascending, the hip point reaches the lowest point of the gravitational phase, and the angular velocity of the hip joint decreases.

Kinetic energy continues to decline until the shoulder point transitions into the anti-gravitational phase (position 53, the third quadrant of the shoulder point’s circular movement). Between positions 50 and 53, while kinetic energy is still decreasing, retroflexion in the shoulder joint begins, accompanied by maximum flexion in the hip joint.

**Figure 7:**
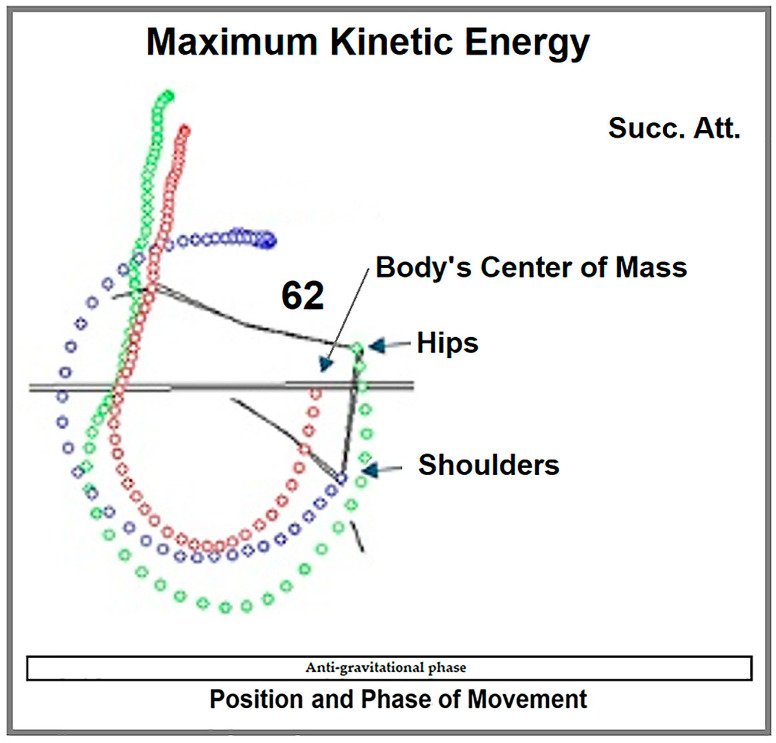
The body’s center of mass and body segments in position 62—kinetic energy at its highest level; Succ. Att.—figure made using the mean values of successful attempts.

After position 53, as the hip point rises above the level of the shoulder point, kinetic energy starts to increase again, initiating a phase of accelerated hip extension and shoulder anteflexion, and maximum kinetic energy is achieved at position 62, driven by the continued increase in the angular velocity of hip extension and shoulder anteflexion. The energy is partially maintained through the next two positions, at which point the center of mass and the hip point are positioned above the bars (Figure 7).

**Figure 8:**
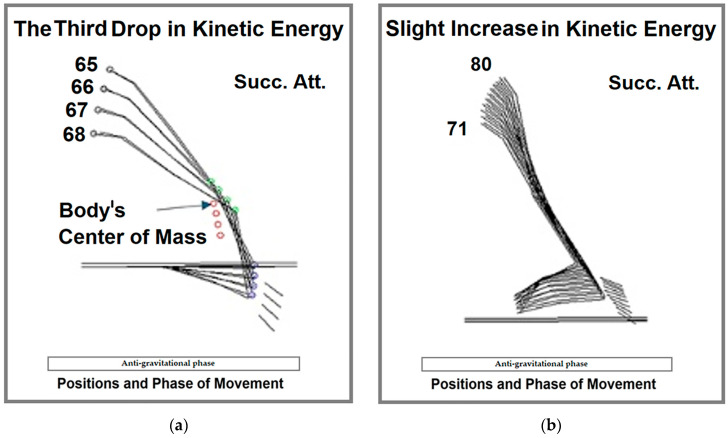
The movement of the body’s center of mass in the x/y plane through positions: (**a**) 65–68—beginning of the steep drop in kinetic energy and (**b**) 71–80—non-support phase; Succ. Att.—figure made using mean values of successful attempts.

Following position 65, as the tips of the feet attain maximum velocity and the hip joint reaches its peak velocity, a sharp drop in kinetic energy occurs. The movement from position 65 to 68 exemplifies the biomechanical principle of transferring kinetic energy from the open end of the kinetic chain (feet) to the closed end (shoulder point) (Figure 8a).

In the final stages of the movement, a minor wave appears—a slight increase in kinetic energy between positions 71 and 80 (Figure 8b). This occurs during the latter part of the non-support phase and the re-establishment of contact with the bars. After re-contacting the bars, kinetic energy decreases steadily until the conclusion of the movement.

As shown in Table 1, translational kinetic energy exhibited a statistically significant difference between successful and unsuccessful attempts from position 56 onward. The average value of translational kinetic energy at position 56 was 338.640 J for successful attempts, compared to 293.616 J for unsuccessful attempts, representing a difference of 45.024 J (15%) in favor of successful attempts. The effect size (ES) using Cohen’s d is 1.37, indicating a large effect and a pronounced difference.

Rotational kinetic energy also displays a statistically significant difference starting from position 57. Successful attempts show an average value of 218.593 J, while unsuccessful attempts average 186.177 J, resulting in a difference of 32.416 J (17%). The ES is 1.24, again indicating a large effect.

Total kinetic energy revealed a statistically significant difference at position 57, with successful attempts averaging 596.541 J compared to 508.694 J for unsuccessful attempts. This difference of 87.847 J (17%) highlights a very large effect size (ES = 1.53), underscoring a pronounced distinction favoring successful attempts.

**Figure 9:**
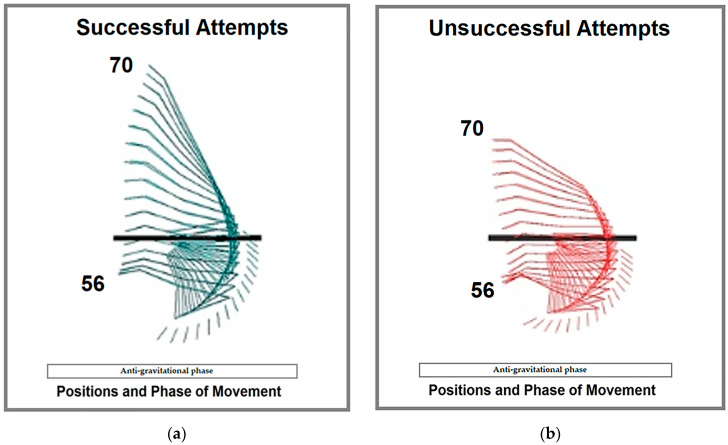
Attempts: (**a**) successful (green stick figures), and (**b**) unsuccessful (red stick figures), positions 56–70.

The *t*-test results confirmed statistically significant differences (*p* < 0.05) between successful and unsuccessful attempts for translational, rotational, and total kinetic energy from positions 56–57 onward (Figure 9). These findings support the conclusion that the observed differences were not due to chance, with greater kinetic energy values consistently associated with successful attempts.

The large ES (Cohen’s d > 0.8) values observed for all kinetic energy variables indicate that the differences are not only statistically significant but also practically meaningful, highlighting their substantial impact on performance outcomes. Furthermore, the percentage differences between successful and unsuccessful attempts, ranging from 15% to 17%, reinforce the importance of these kinetic parameters in determining success, as validated by the *t*-test and effect size results.

## 4. Discussion

The execution of complex gymnastics skills, like the basket to handstand, requires the precise coordination of biomechanical factors and technical execution to achieve successful performance. However, the factors that differentiate successful from unsuccessful attempts in elite-level gymnastics are not fully understood, particularly regarding the role of kinetic energy in movement efficiency and control. Identifying these differences is crucial for optimizing training methods and refining techniques.

The aim of this study was to examine the differences in the kinetic energy of the body’s center of mass between successful and unsuccessful attempts of the basket-to-handstand on the parallel bars performed by an elite gymnast. The main findings of the study revealed significant differences in kinetic energy between successful and unsuccessful attempts, with large ES values for all analyzed variables. These findings can help identify key techniques and movement patterns that contribute to the success of gymnastics elements.

The efficient use of kinetic energy is crucial for the successful execution of gymnastics elements [21]. Recent studies suggest that biomechanical analyses can significantly contribute to optimizing performance techniques, movement stability, efficiency, and precision, particularly in disciplines such as acrobatics and gymnastics [22,23]. These insights can further support achieving better results [24].

The study results highlight that differences in kinetic energy begin at the start of the second phase of movement, the anti-gravitational phase, specifically during the sub-phase of the lifted hang (Figure 9). Regarding trajectory differences, successful attempts were characterized by greater and more consistent kinetic energy values.

Based on these findings, the following observations can be made:

A higher position of the feet and hips at the start of the anti-gravitational phase in successful attempts may indicate better biomechanical optimization of the movement, allowing the gymnast to use gravitational force more effectively to generate kinetic energy. Because of their initial height above the bars, the gymnast has a lot of potential energy at the lowest point of the “basket” posture. During the upward swing, this potential energy which was obtained from the original downward swing is successfully converted into kinetic energy. In order to gain enough height and velocity to return to the handstand posture with ease, this energy conversion is essential.

Kinetic energy is directly influenced by velocity, as it is proportional to the square of velocity. In artistic gymnastics velocity has an important role in successfully performing elements [25]. Greater translational and rotational kinetic energy observed in successful attempts indicates a greater peripheral velocity of body segments (feet, hips, and shoulders) and increased angular velocities in hip extension and shoulder anteflexion, highlighting the critical role of velocity in achieving optimal performance. This is in accordance with the study of Gervais and Dunn [26], who showed that greater vertical velocity induces better performance in the double back salto dismount on parallel bars.

Therefore, greater peripheral velocity values at the tips of the feet, hips, and shoulder point, along with greater angular velocities of hip extension and shoulder anteflexion in successful attempts, indicate better coordination and synchronization of movements. This contributes to improved force transmission through the kinetic chain, enhancing overall movement efficiency [27].

Earlier and faster opening of the kinetic chain: In successful attempts, the opening of the kinetic chain begins earlier and faster due to a more favorable position when entering the second phase (higher hips and feet, smaller shoulder joint angle). This suggests that the gymnast is more successful in initiating and executing movements at critical moments, enabling the more efficient use of accumulated kinetic energy.

Successful attempts demonstrate more proper use of accumulated energy during the gravitational phase, allowing for better performance in the anti-gravitational phase. This indicates that the gymnast has greater control over kinetic energy, optimizing its use to achieve greater velocity and precision.

Greater peripheral velocity values at the tips of the feet, hips, and shoulder point, along with greater angular velocities of hip extension and shoulder anteflexion in successful attempts, indicate better coordination and synchronization of movements. This contributes to improved force transmission through the kinetic chain, enhancing overall movement efficiency.

The results of this study can be directly applied to the training process by focusing on the optimal use of kinetic energy in key movement phases. This includes introducing specific exercises that simulate the transition from the gravitational to the anti-gravitational phase, thereby improving movement efficiency and reducing the risk of injury. In practice, essential pedagogical principles should be followed [28].

Similar to the findings of Wasserberger and associates [8], who demonstrated that throwing velocity in young baseball pitchers largely depends on the efficient transfer of energy between the shoulder and elbow, this study highlights that the efficient use of kinetic energy is critical for the successful execution of gymnastics elements. Although handgrip strength serves as a common link between baseball players and gymnasts [29], from a practical standpoint, significant handgrip strength is crucial, primarily for performance improvement. Additionally, it should not be overlooked that gymnasts perform elements around stationary apparatus for extended periods [29,30,31,32]. Consequently, regarding its contribution as a significant factor in parallel bar performance [33], our findings also indicate that greater kinetic energy in key movement phases leads to improved performance, consistent with the aforementioned studies.

Jones and associates [10] developed a method for evaluating the kinetic energy of athletes using the “run-shuttle” test, which enabled a more precise identification of athlete characteristics based on velocity and kinetic energy. Similarly, our analysis of kinetic energy in gymnastics elements follows comparable principles, where greater kinetic energy values are associated with more successful element execution. This confirms the universal significance of kinetic energy as a key factor in assessing sports performance, whether in dynamic sports like football or in sports with complex coordination demands such as gymnastics. These findings can help enhance training programs to optimize and maximize performance across a range of sports disciplines. The practical implications of these findings can be applied in targeted training processes through specific exercises that simulate transitions between movement phases, improving movement efficiency and reducing injury risks.

This study is limited by its sample size, as it analyzed only one elite gymnast. Future research should include a larger sample size to generalize the results. Additionally, incorporating other complex elements and different apparatus could contribute to a more comprehensive understanding of the role of kinetic energy in gymnastics.

## 5. Conclusions

Based on the analysis, we concluded that successful attempts demonstrate better biomechanical optimization and more efficient use of kinetic energy during the execution of exercises. The results of this study indicate that an earlier entry into the second phase of movement, with a higher position of the feet and hips and a smaller shoulder joint angle, allows for a more efficient initiation of the open end of the kinetic chain. Consequently, this results in greater peripheral and angular velocities, which are crucial for the successful execution of gymnastics elements.

The findings of this study complement existing knowledge about kinetic energy in sports and confirm the importance of energy transfer through the kinetic chain, especially in gymnastics, where the demand for biomechanical analysis is increasing to enhance execution techniques. This knowledge will further contribute to improving performance fluidity while minimizing the risk of injury. Beyond gymnastics, these findings are also confirmed in other sports, highlighting the universality of biomechanical principles that can be applied across various disciplines to optimize performance.

## Figures and Tables

**Table 1:** Comparison of kinetic energy variables between successful and unsuccessful attempts.

Variable	KP	Successful [J]	Unsuccessful [J]	Diff [J]	*t*-Test	*p*	∆ (%)	ES
KEtrans	56	338.640	293.616	45.024	2.412	0.03	15%	1.37 ***
KErot	57	218.593	186.177	32.416	2.222	0.04	17%	1.24 ***
KEtotal	57	596.541	508.694	87.847	2.636	0.02	17%	1.53 ***

Legend: KEtotal—total kinetic energy; KEtrans—kinetic energy of translational motion; KErot—kinetic energy of rotational motion; KP—position where statistically significant difference begins; J—joules; Diff—numerical difference between arithmetic means; *t*-Test—numerical value of *t*-test; *p*—statistical significance of *t*-test; ∆ (%)—percent difference between successful and unsuccessful performances; difference is very pronounced and significant ***; ES—Effect Size.

## Data Availability

The data provided in this study can be obtained upon request from the corresponding author.
